# ABC transporter FtsABCD of *Streptococcus pyogenes *mediates uptake of ferric ferrichrome

**DOI:** 10.1186/1471-2180-5-62

**Published:** 2005-10-14

**Authors:** Tracey S Hanks, Mengyao Liu, Michael J McClure, Benfang Lei

**Affiliations:** 1 Department of Veterinary Molecular Biology, Montana State University, Bozeman, Montana 59717, USA

## Abstract

**Background:**

The *Streptococcus pyogenes *or Group A *Streptococcus *(GAS) genome encodes three ABC transporters, namely, FtsABCD, MtsABC, and HtsABC, which share homology with iron transporters. MtsABC and HtsABC are believed to take up ferric (Fe^3+^) and manganese ions and heme, respectively, while the specificity of FtsABCD is unknown.

**Results:**

Recombinant FtsB, the lipoprotein component of FtsABCD, was found to bind Fe^3+ ^ferrichrome in a 1:1 stoichiometry. To investigate whether FtsABCD transports Fe^3+ ^ferrichrome, GAS isogenic strains defective in lipoprotein gene *ftsB *and permease gene *ftsC *were generated, and the effects of the mutations on uptake of Fe^3+ ^ferrichrome were examined using radioactive ^55^Fe^3+ ^ferrichrome. FtsB was produced in the wild-type strain but not in the *ftsB *mutant, confirming the *ftsB *inactivation. While wild-type GAS took up 3.6 × 10^4 ^Fe^3+ ^ferrichrome molecules per bacterium per min at room temperature, the *ftsB *and *ftsC *mutants did not have a detectable rate of Fe^3+ ^ferrichrome uptake. The inactivation of *ftsB *or *ftsC *also decreased ^55^Fe^3+ ^ferrichrome uptake by >90% under growth conditions in the case of limited uptake time. Complementation of the *ftsB *mutant with a plasmid carrying the *ftsB *gene restored FtsB production and ^55^Fe^3+ ^ferrichrome association at higher levels compared with the parent strain. The inactivation of *mtsA *and *htsA *and Fe-restricted conditions enhanced the production of FtsB and Fe^3+ ^ferrichrome uptake.

**Conclusion:**

The FtsB protein bound Fe^3+ ^ferrichrome, and inactivation of *ftsB *or *ftsC*, but not *htsA *or *mtsA*, diminished Fe^3+ ^ferrichrome uptake, indicating that FtsABCD, but not HtsABC and MtsABC, is the transporter that takes up Fe^3+ ^ferrichrome in GAS. Fe acquisition systems are virulence factors in many bacterial pathogens and are attractive vaccine candidates. The elucidation of the FtsABCD specificity advances the understanding of Fe acquisition processes in GAS and may help evaluating the GAS Fe acquisition systems as vaccine candidates.

## Background

Ferric iron (Fe^3+^), the stable iron form in an oxidative environment, has extremely low solubility in water under physiological conditions, and mammalian hosts thus do not have sufficient free Fe^3+ ^to support bacterial growth [1]. The major sources of iron in vivo for bacteria are host heme-proteins and other iron complexes [1,2]. Many bacterial pathogens secrete low-molecular-weight iron chelators called siderophores to assimilate iron from host environments [3]. Ferrisiderophores formed are then transported across the cytoplasmic membrane by specific ATP-binding cassette (ABC) type transporters. ABC transporters consist of a solute-binding protein, a membrane protein (permease) encoded by one or two genes, and an ATPase [4]. The solute-binding proteins are located in the periplasmic space in Gram-negative bacteria and are lipoproteins in Gram-positive organisms. Siderophores can be divided into several types based on chemical structures [3]. Ferrichrome [5] belongs to the hydroxamate type.

*Streptococcus pyogenes *or Group A *Streptococcus *(GAS) is an important Gram-positive human pathogen causing both invasive and non-invasive infections [6]. Non-invasive infections, including pharyngitis, and post-infection sequelae, such as acute rheumatic fever, rheumatic heart disease, and glomerulonephritis, result in substantial morbidity and economic loss globally. Invasive GAS infections, such as necrotizing fasciitis and streptococcal toxic shock syndrome, are associated with high mortality rates. GAS can take up heme from hemoglobin and haptoglobin-hemoglobin complexes [7]. Exogenously-supplied heme and host heme proteins (hemoglobin, myoglobin, and catalase), but not iron-loaded transferrin and lactoferrin, support in vitro growth of GAS under iron-restricted conditions [8].

Iron acquisition processes in GAS are poorly understood, although progress has recently been made by us [9-11] and other groups [12-14]. GAS genomes [15-17] encode three ABC transporters, namely, HtsABC [9-11] or SiaABC [12], MtsABC [13,14], and one encoded by *spy0383 *to *spy0386 *[18] (designated FtsABCD), which are homologues of ABC transporters involved in iron acquisition. HtsABC and the cell-surface protein Shp [9-12] are believed to make up the machinery for heme acquisition. The lipoprotein component MtsA of MtsABC binds Fe^3+^, Zn^2+^, and Cu^2+ ^[13], and MtsABC is important for acquisition of Mn^2+ ^and Fe^3+ ^[14]. The transcription of *fts*ABCD is up-regulated under iron-restricted conditions [18]. However, recombinant FtsB was heme-free and did not contain Fe, Mn, or Zn [10]. The specificity of FtsABCD is thus not known. It is not known whether GAS can use ferrisiderophores such as ferric ferrichrome (Fe^3+ ^ferrichrome) as an iron source.

We found that recombinant FtsB bound  Fe^3+^ ferrichrome, suggesting that FtsABCD is involved in the acquisition of Fe^3+ ^ferrichrome. To test this hypothesis, GAS isogenic strains defective in lipoprotein gene *ftsB *and permease gene *ftsC *were generated, and the *ftsB *and *ftsC *inactivation dramatically decreased the uptake of Fe^3+ ^ferrichrome, indicating that FtsABCD is the transporter that takes up Fe^3+ ^ferrichrome in GAS.

## Results

### Binding of Fe^3+ ^ferrichrome by FtsB

**Figure 1:**
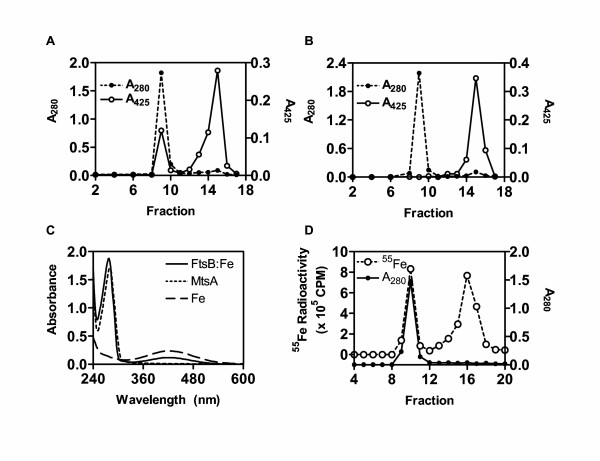
**Binding of Fe^3+ ^ferrichrome to FtsB**. (A) Co-migration of Fe^3+ ^ferrichrome with FtsB. FtsB (0.3 ml of 0.18 mM) was incubated with 0.9 mM Fe^3+ ^ferrichrome for 20 min at room temperature, loaded onto a Sephadex G-25 column (1.5 × 18 cm), and eluted with 20 mM Tris-HCl buffer, pH 8.0. Eluant was collected as fractions of 0.7 ml. A_280 _and A_425 _of each fraction were used to monitor FtsA and Fe^3+ ^ferrichrome, respectively. (B) MtsA, as a control, did not bind Fe^3+ ^ferrichrome. The experiment was performed under the conditions as those in panel A. (C) The absorption spectra of the protein peaks (fraction 9) in panel A (solid curve) and panel B (short dashed curve) and free Fe^3+ ^ferrichrome (long dashed curve). The three samples contained 46 μM FtsB, 45 μM MtsA, and 100 μM Fe^3+ ^ferrichrome, respectively. (D) Co-migration of partially ^55^Fe-labeled Fe^3+ ^ferrichrome with FtsB. Fe^3+ ^ferrichrome contained radioactive ^55^Fe^3+ ^at 1.1% of total Fe^3+^. A_280 _and ^55^Fe^3+ ^radioactivity of each fraction are presented.

The specificity of an ABC transporter is believed to depend on the binding specificity of its solute binding protein. Purified recombinant FtsB did not contain free metal ions, suggesting that FtsABCD does not target free metal ions [10]. To test whether FtsABCD targets ferrisiderophores, the ability of FtsB to bind Fe^3+ ^ferrichrome was examined by gel filtration. FtsB was incubated with excess Fe^3+^ ferrichrome and separated from free Fe^3+ ^ferrichrome by chromatography on a Sephadex G-25 column. Fe^3+ ^ferrichrome has an absorption peak at 425 nm and its elution profile was monitored by measuring A_425 _of each fraction. The elution profile displayed two A_425 _peaks (Fig. 1A). One co-migrated with the FtsB peak, which was localized by A_280_, and the other corresponded to free Fe^3+ ^ferrichrome (Fig. 1A). When FtsB was replaced with MtsA in a control experiment, there was only one A_425 _peak corresponding to free Fe^3+ ^ferrichrome, and no absorbance at 425 nm was associated with the protein peak (Fig. 1B). To check whether the species associated with FtsB was Fe^3+ ^ferrichrome, the absorption spectra of the FtsB and MtsA peaks and free Fe^3+ ^ferrichrome were compared. In addition to the protein absorption peak at A_280_, the FtsB sample had an absorption peak at 425 nm which was identical to that of free Fe^3+ ^ferrichrome (Fig. 1C). As expected, the MtsA sample only had the protein peak (Fig. 1C). These results indicate that FtsB, but not MtsA, bound Fe^3+^ ferrichrome. The FtsB sample was found to have 1.08 Fe^3+ ^ferrichrome per FtsB molecule on the basis of protein content and extinction coefficient of Fe^3+ ^ferrichrome at 425 nm, indicating a 1:1 binding stoichiometry.

If FtsB binds Fe^3+ ^ferrichrome, Fe^3+ ^should co-migrate with the protein on the G-25 column. To test this idea, Fe^3+ ^ferrichrome containing 1.1% ^55^Fe^3+ ^of total Fe^3+ ^was used to repeat the gel filtration experiment, and ^55^Fe^3+ ^radioactivity was monitored. As expected, one of two ^55^Fe^3+ ^peaks co-migrated with the FtsB peak, and the other peak corresponded to free Fe^3+ ^ferrichrome (Fig. 1D). On the basis of ^55^Fe^3+ ^percentage of total iron and specific activity and protein content, the FtsB peak fraction contained 37 μM FtsB and 36 μM Fe, consistent with the 1:1 binding stoichiometry determined above. These results confirmed that FtsB binds Fe^3+ ^ferrichrome in a 1:1 molar ratio.

### Effects of *ftsB*, *ftsC*, *mtsA*, and *htsA* inactivation on Fe^3+ ^ferrichrome uptake

**Figure 2:**
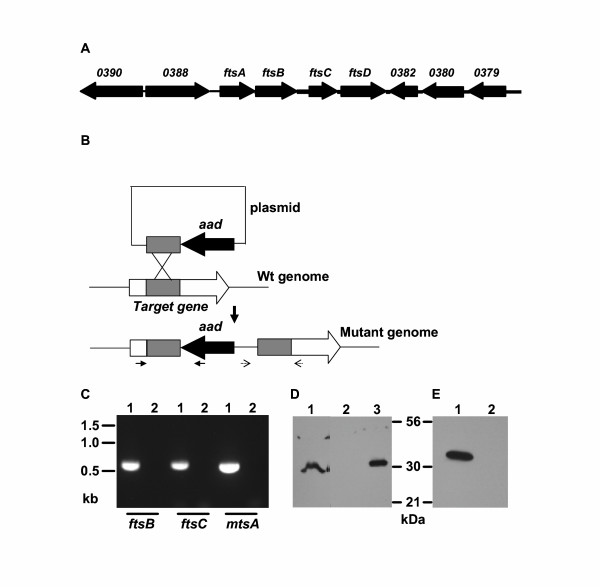
**Insertional inactivation of *ftsB*, *ftsC*, and *mtsA***. (A) Schematic showing the arrangement of *ftsABCD *genes and their neighbors in the MGAS5005 genome. The *ftsA*, *ftsB*, and *ftsCD *genes encode ATP-binding protein, lipoprotein, and permease, respectively. The numbers above the other arrows are the *spy *numbers assigned to the corresponding open reading frames in the M1 genome sequence (15). (B) Schematic for insertional gene inactivation. The paired solid or dotted arrows under the mutant genome indicate the locations of primers used to confirm the disruption of the gene using PCR and DNA sequencing. (C) PCR confirmation of insertional inactivation of *ftsB*, *ftsC*, and *mtsA*. The picture shows agarose gel analysis of PCR reactions using mutant (lanes labeled by 1) or wild type (lanes labeled by 2) genomic DNA as template and primers at the locations indicated by the solid arrows under the mutant genome in panel B. (D) Western blot showing the absence of FtsB in the *ftsB *mutant strain (lane 2) and the presence of FtsB in the wild-type (lane 1) and *ftsB*/pCMV*ftsB *(lane 3) strains. Proteins from 5 × 10^8 ^wild-type cells, 5 × 10^8 ^*ftsB *mutant cells, and 4 × 10^6 ^*ftsB*/pCMV*ftsB *were probed with FtsB-specific mouse antiserum. The amount of *ftsB*/pCMV*ftsB *cells used was 1/125 of that of the wild-type cells. (E) Western blot showing the presence of MtsA in the wild-type strain (lane1) and the absence of MtsA in the *mtsA *mutant strain (lane 2). Proteins from 6 × 10^6 ^wild-type cells and 3 × 10^8 ^*mtsA *mutant cells were probed with MtsA-specific mouse antiserum.

**Figure 3:**
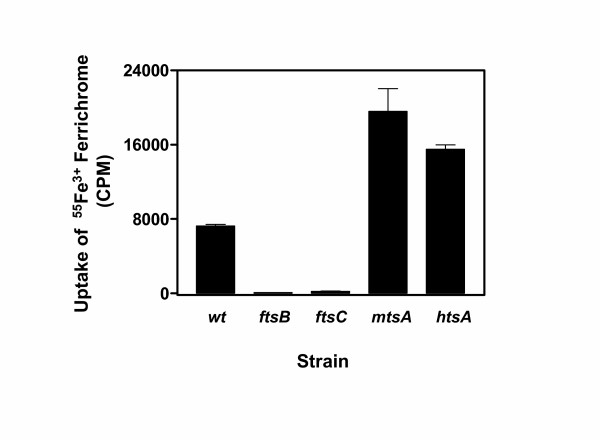
**Effects of *ftsB*, *ftsC*, *mtsA*, and *htsA *inactivation on ^55^Fe^3+ ^ferrichrome uptake**. Wild-type and mutant strains were grown to OD_600 _of 0.4 in 10 ml of THY and THY/spectinomycin, respectively. The bacterial pellets were washed with 1.0 ml of THY and incubated with 0.16 μM ^55^Fe^3+ ^ferrichrome 1.0 ml of THY at room temperature for 1 h. A triplet of 0.2 ml samples were taken from each mixture, and bacteria were pelleted and washed twice with 0.4 ml of THY. ^55^Fe^3+ ^radioactivity associated with the bacteria was measured. Presented are the mean values ± SD of ^55^Fe^3+ ^radioactivity associated with the bacteria in a representative of three experiments.

**Figure 4:**
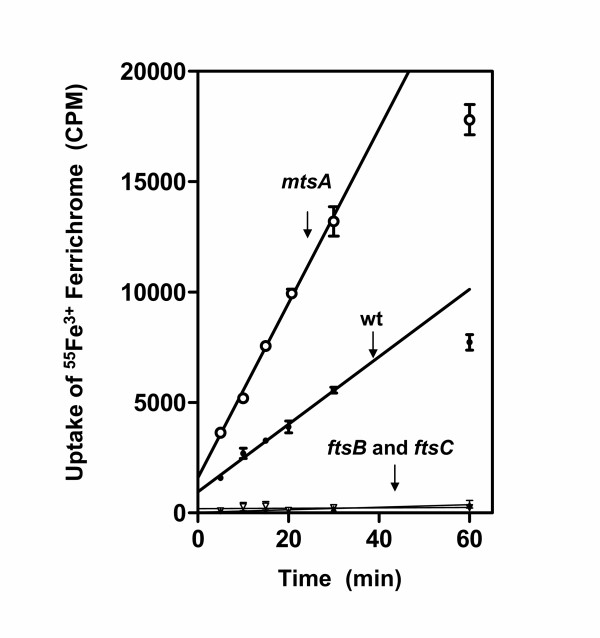
**The time course of ^55^Fe^3+ ^ferrichrome uptake for *mtsA *(open circles), *ftsB *(open triangles), and *ftsC *(solid triangles) mutant and wild-type (solid circles) GAS strains**. Each strain harvested at the mid-exponential growth phase from 40 ml culture, washed with 10 ml of THY, and incubated with 0.16 μM ^55^Fe^3+ ^ferrichrome in 4 ml of THY at room temperature. A triplet of 0.2 ml samples were taken from each mixture at the indicated times, and bacteria were immediately pelleted and washed twice with 0.4 ml of THY. Presented are the mean values ± SD of ^55^Fe^3+ ^radioactivity associated with the bacteria in a representative of three experiments.

The binding results described above suggest that FtsABCD can acquire Fe^3+ ^ferrichrome. To test this hypothesis, *ftsB*, *ftsC*, and *mtsA *(control) were first inactivated by insertional inactivation (Fig. 2). Inactivation was confirmed by PCR (Fig. 2C) and DNA sequencing. Western blotting analysis detected FtsB (Fig. 2D) and MtsA (Fig. 2E) in the wild-type strain but not in the corresponding mutant strain. These results indicate that *ftsB *and *mtsA *were indeed inactivated. The *ftsB, ftsC, mtsA*, and *htsA *mutants (construction of the *htsA *mutant will be described elsewhere) and parent strains were compared in the uptake of ^55^Fe^3+ ^ferrichrome. Pilot experiments indicated that the wild-type strain could take up ^55^Fe^3+ ^ferrichrome at both room temperature (24°C) and 37°C. The uptake was thus performed at room temperature for convenience. GAS cells harvested from exponential growth phase were incubated with 0.16 μM ^55^Fe^3+ ^ferrichrome at room temperature for 1 h, and ^55^Fe^3+ ^radioactivity associated with bacteria was determined. The *ftsB *and *ftsC *inactivation diminished the uptake of Fe^3+ ^ferrichrome (Fig. 3), while the *mtsA *and *htsA *inactivation did not abolish but enhanced the uptake. To further examine the uptake of Fe^3+ ^ferrichrome, ^55^Fe^3+ ^radioactivity taken up by bacteria as a function of incubation time was determined. ^55^Fe^3+ ^radioactivity with wild-type GAS increased linearly with time up to 30 min with a slope of 153 cpm/min (Fig. 4). The value of the slope could be translated into an uptake rate of 3.6 × 10^4 ^Fe^3+ ^ferrichrome molecules per min per wild-type GAS bacterium. In contrast, the *ftsB *and *ftsC *mutants did not have a detectable rate of Fe^3+ ^ferrichrome uptake (Fig. 4). The rate of uptake in the *mtsA *mutant was 2.6 times as that in the parent strain (Fig. 4). These results indicate that FtsABCD, not MtsABC and HtsABC, mediates the uptake of Fe^3+ ^ferrichrome.

### Complementation

**Figure 5:**
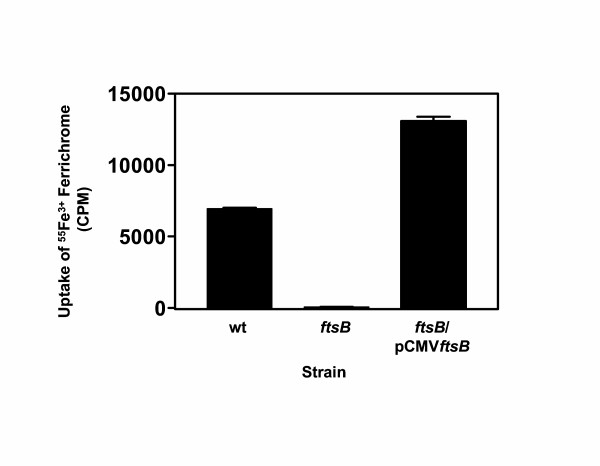
**Complementation of the *ftsB *mutant**. Plasmid pCMV*ftsB *carrying the *ftsB *gene was introduced into the *ftsB *mutant strain to obtain *ftsB*/pCMV*ftsB*. The uptake experiment was performed exactly as in Fig. 3. Presented are the mean values ± SD of ^55^Fe^3+ ^radioactivity associated with the bacteria.

To test whether the effect of the *ftsB *inactivation on the uptake of Fe^3+ ^ferrichrome was due to the absence of FtsB, a complementing strain,*ftsB mutant/*pCMV*ftsB*, was constructed to carry plasmid pCMV*ftsB *with the *ftsB *gene. The intensity of FtsB band of the complemented strain in Western blotting analysis in Fig. 2D was 7/10 of that of the wild-type strain, and the amount of the complemented strain cells used was 1/125 of that of the wild-type cells, indicating that FtsB was produced in *ftsB mutant/*pCMV*ftsB *at a level as 88 times as that in the wild-type strain. Wild-type, *ftsB *mutant, and *ftsB *mutant*/*pCMV*ftsB *cells harvested in the exponential growth phase were incubated with 0.16 μM ^55^Fe^3+ ^ferrichrome at 24°C for 1 h, and ^55^Fe^3+ ^radioactivity associated with the bacteria was determined. ^55^Fe^3+ ^radioactivity of *ftsB mutant/*pCMV*ftsB *was 1.9 and 207 times higher than those of the wild-type and *ftsB *mutant cells, respectively (Fig. 5), suggesting that in trans expression of *ftsB *in the *ftsB *mutant restored the association of Fe^3+ ^ferrichrome. The results support that FtsABCD targets Fe^3+ ^ferrichrome.

### Effect of Fe^3+^ ferrichrome on GAS growth

Since FtsABCD is involved in the uptake of Fe^3+ ^ferrichrome, Fe^3+ ^ferrichrome should be an iron source of GAS. This idea was tested by comparing GAS growth curves in THY treated with Chelex 100 to remove metal ions and supplemented with MgCl_2 _(DTHYMg) in the absence and presence of Fe^3+^ ferrichrome. The growth curve in the presence of 10 μM Fe^3+^ ferrichrome shifted to the left by about 40 min compared with that in the absence of Fe^3+^ ferrichrome under otherwise identical conditions. This small but repeatable stimulatory effect suggests that GAS can use Fe^3+^ ferrichrome as an iron source. Fe^3+^ ferrichrome had similar stimulatory effect on the growth of the mutants as that on the growth of the wild type strain, suggesting that either residual uptake of Fe^3+^ ferrichrome in the *ftsB *and *ftsC *mutants were enough to induce the stimulatory effect or an additional process was involved in the uptake of the iron extracted from Fe^3+^ ferrichrome under the growth conditions.

### Effects of *ftsB* and *ftsC* inactivations on the uptake of Fe^3+ ^ferrichrome under growth conditions

**Figure 6:**
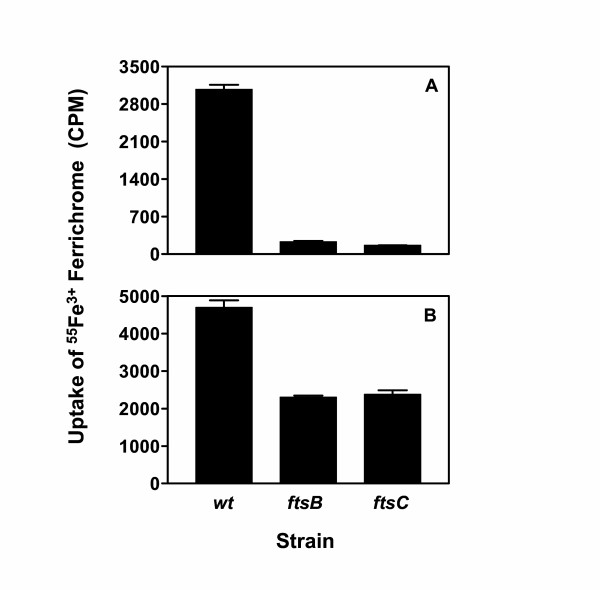
**Effects of *ftsB *and *ftsC *inactivation on ^55^Fe^3+ ^ferrichrome uptake under growth conditions**. (A) Uptake for 30 min. ^55^Fe^3+ ^ferrichrome (0.16 μM) was added into cultures of the wild-type and mutant strains at 37°C in THY and THY supplemented with spectinomycin, respectively, when OD_600 _was 0.3. A triplet of 1 ml samples were taken from each culture 30 min later, and bacteria were immediately pelleted and washed twice with 0.4 ml of THY. Presented are the mean values ± SD of ^55^Fe^3+ ^radioactivity associated with the bacteria. (B) Uptake for 3 h. ^55^Fe^3+ ^ferrichrome (0.16 μM) was added into the wild-type and mutant cultures at 37°C when OD_600 _was 0.2, and, 3 h later, the samples were processed as in panel A for determination of ^55^Fe^3+ ^radioactivity.

To examine why the stimulatory growth effect of Fe^3+^ ferrichrome was still observed in the *ftsB *and *ftsC *mutants, ^55^Fe^3+ ^ferrichrome uptake was examined under growth conditions. ^55^Fe^3+ ^ferrichrome was added into the cultures of wild-type, *ftsB*, and *ftsC *strains at mid-exponential growth phase, and, 30 min later, ^55^Fe^3+ ^activity associated with bacteria was determined. ^55^Fe^3+ ^radioactivity of *ftsB *and *ftsC *mutant cells were only 7.5% and 5.3% of that of wild-type cells, respectively (Fig. 6A), indicating that the *ftsB *and *ftsC *inactivation also dramatically diminished uptake of Fe^3+ ^ferrichrome under growth conditions. However, ^55^Fe^3+ ^radioactivity of *ftsB *and *ftsC *cells grown for 3 h after the addition of ^55^Fe^3+ ^ferrichrome were about 50% of that of wild-type cells (Fig. 6B). These results suggest that there could be an additional acquisition process which could assimilate ^55^Fe^3+ ^from its ferrichrome complex.

### Effects of *mtsA* and *htsA* inactivation and Fe-restricted conditions on Fe^3+ ^ferrichrome uptake and *FtsB* production

**Figure 7:**
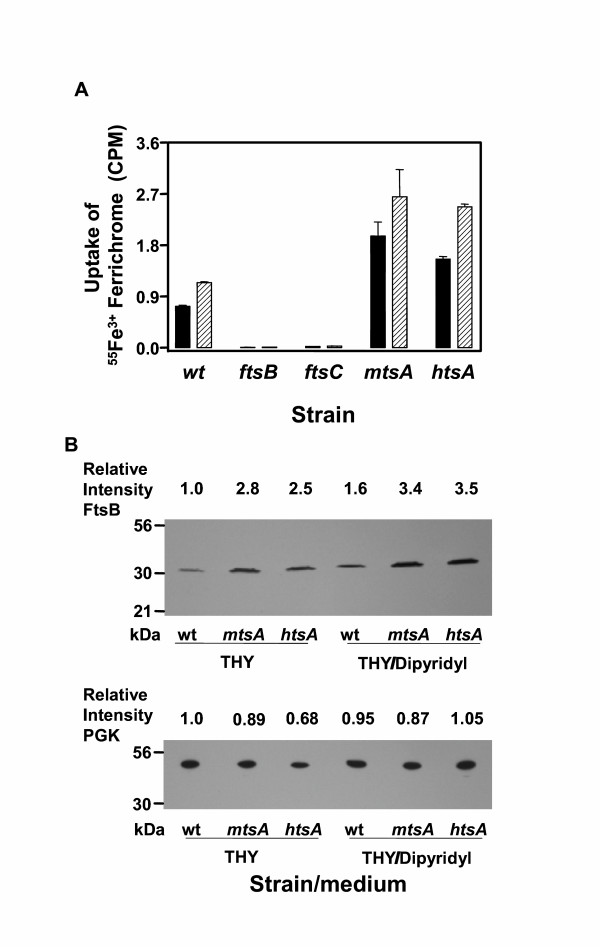
**Effects of Fe-restricted conditions and *mtsA *and *htsA*inactivation on Fe^3+ ^ferrichrome uptake and FtsB production**. (A). ^55^Fe^3+ ^radioactivity uptake by wild-type (wt), *ftsB*, *ftsC*, *mtsA*, and *htsA *mutant strains grown in THY in the absence (solid bars) or presence (slashed bars) of 0.3 mM 2,2'-dipyridyl. The experiment was performed as in Fig. 3. (B) Western blots showing the relative levels of FtsB and PGK as a control in the strains grown under the same conditions as in panel A. Proteins from 1 × 10^8 ^bacteria of each strain were probed with mouse anti-FtsB and anti-PGK antisera. The relative levels of FtsB and PGK were obtained by dividing the intensity of each band by the intensity of the FtsB or PGK band of the wild-type strain.

To further examine the factors to affect Fe^3+ ^ferrichrome uptake, the effects of *mtsA *and *htsA *inactivation and Fe-restricted conditions on FtsB production and Fe^3+ ^ferrichrome uptake were investigated. Inactivation of *htsA *and *mtsA *increased ^55^Fe^3+ ^ferrichrome uptake by more than 100% (Fig. 7A). Wild-type, *htsA*, and *htsA *mutant cells harvested from THY containing 2,2'-dipyridyl took up 30%–50% more ^55^Fe^3+ ^ferrichrome than those from THY without 2,2'-dipyridyl (Fig. 7A). Western blotting analysis indicated higher levels of FtsB in the *htsA *and *mtsA *mutants compared with the wild-type strain (Fig. 7B). Higher levels of FtsB were also detected in all strains grown in the presence of 2,2'-dipyridyl compared with those grown in the absence of 2,2'-dipyridyl (Fig. 7B). A control protein, phosphoglycerate kinase (PGK), had similar expression levels under all the conditions (Fig. 7B). The relative intensities of the FtsB bands in the Western blot correlated well with the relative uptake of ^55^Fe^3+ ^ferrichrome under the same conditions. These results indicate that FtsB production was enhanced in the *htsA *and *mtsB *mutants and under Fe-restricted conditions, further supporting the role of FtsABCD in Fe acquisition.

## Discussion

Evidences from this study indicate that FtsABCD is the transporter that takes up Fe^3+ ^ferrichrome in GAS. The evidences include the binding of Fe^3+ ^ferrichrome to FtsB, the effect of the insertional disruption of *ftsB *or *ftsC *on uptake of ^55^Fe^3+ ^ferrichrome, and the non-involvement of MtsABC and HtsABC in Fe^3+ ^ferrichrome uptake. Complementation data of the *ftsB *mutant with the *ftsB *gene expressed in trans indicated that the effect of *ftsB *inactivation was due to the lack of FtsB. MtsABC [13,14] and HtsABC [9-12] target free Fe^3+ ^and heme, respectively. The specificity of the FtsABCD transporter elucidated in this study resolved the last piece of the puzzle regarding the roles of ABC transporters in Fe acquisition in GAS.

Uptake time was critical to the effects of *ftsB *and *ftsC *inactivation on ^55^Fe^3+ ^activity associated with bacteria under growth conditions. The inactivation had a dramatic effect (>90% decrease in ^55^Fe^3+ ^activity compared with wild-type strain) when uptake was performed for only 30 min. This decrease was reduced to about 50% when uptake was performed for 3 h. Although the reasons for this reduction are not known, the reduction is unlikely due to the existence of another transporter for Fe^3+ ^ferrichrome. The other uptake results are not consistent with the existence of another Fe^3+^ ferrichrome transporter. Another possible reason is that ^55^Fe^3+ ^ferrichrome exchanged its ^55^Fe^3+ ^with another ferric complex in THY, resulting in a non-ferrichrome ^55^Fe^3+ ^complex that could be taken up by another transporter. GAS has a putative secreted Fe binding protein (Spy1063). It is not known whether this protein can extract Fe^3+ ^from Fe^3+ ^ferrichrome.

The transcription of *ftsABCD *is up-regulated under Fe-reduced conditions [18]. Consistent with this observation, Fe-restricted conditions enhanced the production of FtsB and uptake of Fe^3+ ^ferrichrome, further supporting the role of FtsABCD in Fe acquisition. Inactivation of *htsA *and *mtsA *also enhanced FtsB production and Fe^3+ ^ferrichrome uptake. Apparently, all the three ABC transporters contributed to Fe^3+ ^acquisition in GAS grown in THY, and inactivation of either *mtsA *or *htsA *might result in lower intracellular Fe levels and, in turn, enhanced the expression of *ftsABCD*. The results also suggest that the expression of *ftsABCD*, *htsABC*, and *mtsABC *is coordinately regulated.

Some *Enterococcus faecium *clinical strains do not produce siderophores but can acquire iron using exogenous siderophores produced by other bacteria living in the same habitats [19]. GAS is not known to produce siderophores, and GAS genomes [15-17] do not have genes encoding homologues of siderphore-production systems. We could not detect siderophore production in GAS under iron-restricted conditions. Therefore, GAS may not produce siderophors. However, GAS takes up Fe^3+ ^ferrichrome, suggesting that GAS could acquire Fe^3+ ^by using siderophores produced by other bacteria in the pharynx and skin, the noninvasive GAS infection sites.

Fe acquisition systems are virulence factors in many bacterial pathogens [20-24] and are attractive vaccine targets [25-30]. Elucidation of the specificities of the Fe transporters in GAS will facilitate determination of their relative importance in various infections and choose appropriate animal infection models to evaluate their efficacy as vaccine candidates. For examples, HtsABC could be more important in invasive GAS infection since heme should be the Fe source, and FtsABCD could be important in non-invasive infections because exogenous ferric siderophore complexes should be available.

In summary, we found that FtsB bound Fe^3+ ^ferrichrome and that *ftsB *or *ftsC *inactivation dramatically decreased Fe^3+ ^ferrichrome uptake. The results indicate that FtsABCD is the transporter that acquires Fe^3+ ^ferrichrome in GAS.

## Methods

### Materials

Iron chelating agent 2,2'-dipyridyl was obtained from Aldrich. Sephadex G-25, iron-free ferrichrome A from *Ustilago sphaerogena*, Chelex 100, and other chemicals were purchased from Sigma (St. Louis, MO). ^55^Fe^3+^-labeled ferric chloride was purchased from RI Consultants LLC (Hudson, NH). Purified recombinant FtsB (Spy0385) and MtsA were prepared as described previously [10,31].

### Mouse antisera

Five female outbred CD-1 Swiss mice (4- to 6-week-old) (Charles River Laboratories, Wilmington, MA) were immunized subcutaneously with 50 μg of recombinant FtsB, MtsA, or PGK suspended in 200 μL of saline emulsified in 44 μL of monophosphoryl lipid A-synthetic trehalose dicorynomycolate adjuvant (Corixa, Hamilton, MT). Mice were boosted at weeks 2 and 4. Immune sera were collected 5 days after the second boost.

### Bacterial strains and growth

Serotype M1 GAS strain MGAS5005 has been described previously [32]. MGAS5005 and its isogenic mutants were grown routinely at 37°C in 5% CO_2 _in Todd-Hewitt broth (Difco Laboratories, Detroit, MI) supplemented with 0.2% yeast extract (THY). Spectinomycin (150 mg/L) was added into THY for mutant strains. Iron-restricted conditions were achieved by adding 0.3 mM 2,2'-dipyridyl into THY and by treating THY with the chelating resin Chelex 100 and supplementing it with 0.4 mM MgCl_2 _(DTHYMg). Tryptose agar with 5% sheep blood (Becton Dickinson, Cockeysville, MD) and THY agar were used as solid media.

### Binding of Fe^3+ ^ferrichrome to FtsB

Gel filtration was used to detect the binding of Fe^3+ ^ferrichrome to FtsB. FtsB (0.3 ml of 0.18 mM) was incubated with 0.9 mM Fe^3+ ^ferrichrome for 20 min at room temperature, loaded onto a Sephadex G-25 column (1.5 × 18 cm), and eluted with 20 mM Tris-HCl buffer, pH 8.0. Eluant was collected as fractions of 0.7 ml. The absorbance of each fraction attributable to protein and Fe^3+ ^ferrichrome was measured. MtsA as a negative control was similarly tested for the binding of Fe^3+ ^ferrichrome. The experiment was repeated with Fe^3+ ^ferrichrome in which 1.1% of total Fe^3+ ^was ^55^Fe, and A_280 _and ^55^Fe^3+ ^radioactivity of each fraction were measured. ^55^Fe^3+ ^radioactivity was measured using a window of 0–6 keV with a Packard 1500 Tri-Carb Liquid Scintillation Analyzer.

### GAS isogenic mutants

MGAS5005 isogenic mutants defective in *ftsC*, *ftsB*, or *mtsA *were generated by insertional inactivation (Fig. 2). An internal ~400-bp fragment of each gene was PCR-amplified using MGAS5005 genomic DNA as template and the primers listed in Table 1. The PCR products were digested with *Nco*I and ligated to pFW*aad *at the *Nco*I site to yield suicide plasmids. The orientation of the fragments was determined by DNA sequencing, and the suicide plasmids in which the orientation of the fragments was opposite to that of the *aad *gene were chosen for generating the mutants. To obtain pFW*aad*, the spectinomycin-resistant gene *aad *[33] was amplified with primers 5'-AGTGTCGACTATAACTAATAACGTAACGTG-3' and 5'-ACCATGGGAATTCTATAATTTTTTTAATCTGTTATTTA-3' and cloned into pFW14 [34] at the *Sal*I and *Nco*I restriction sites.

Each suicide plasmid was introduced into MGAS5005 by electroporation at 1.8 kV and 400 Ω. One ml of THY was added into the sample immediately after electroporation. The sample was incubated at 37°C for 2 h and plated on THY agar plates supplemented with 150 mg of spectinomycin per liter to select insertional mutants. The plates were incubated in 5% CO_2 _at 37°C for two days, and the colonies obtained were screened by PCR analysis using the primers listed in Table 1. Gene interruptions were then confirmed by sequencing the PCR products.

### Construction of *ftsB*-complementing plasmid pCMV*ftsB*

Plasmid pJRS525 [35] was modified to replace the spectinomycin-resistant gene with the chloramphenicol-resistance gene. A 2545-bp fragment without the spectinomycin-resistant gene was amplified from pJRS525 with primers 5'-TCGTGGATCC AAGCTTCACCATGG-3' and 5'-AAGAATTCCTTGCATAGACTTTCGTCAG-3'. The fragment containing the chloramphenicol-resistant gene was amplified from pFW14 [23] with primers 5'-GGAATTCCGGATGCATATGCATG-3' and 5'-GTCCGGATCCTCGAGCTCTAGATC-3'. The PCR products were digested with *BamH*I and *EcoR*I and ligated together to yield plasmid pCMV. A DNA fragment containing *ftsB *and its ribosome-binding site was amplified from MGAS5005 using primers 5'-CGGATCCAATAACTTTATTCTAGGAGAATTAG-3' and 5'-AGGGATCCTTAGTTTTCACTTGATAAGATTG-3'. The PCR product was digested with *BamH*I and ligated into pCMV at the *BamH*I site, yielding pCMV*ftsB *containing *ftsB*. The cloned gene was sequenced to rule out spurious mutations and confirm the desired orientation. The resulting pCMV*ftsB *was introduced into the *ftsB *mutant by electroporation, and the complement strain (designated *ftsB*/pCMV*ftsB*) was selected by spectinomycin and chloramphenicol and confirmed for the existence of pCMV*ftsB *by colony PCR using primers 5'-CAATTTCACACAGGAAACAGC-3' (pCMV-specific) and 5'-AGGGATCCTTAGTTTTCACTTGATAAGATTG-3' (*ftsB*-specific)

### Uptake of Fe^3+ ^ferrichrome by GAS

Fe^3+ ^ferrichrome uptake by GAS was monitored using radioactive ^55^Fe^3+ ^ferrichrome under non-growth and growth conditions. Under the non-growth conditions, wild-type MGAS5005 and its *ftsB*, *ftsC*, or *mtsA *mutant strains were harvested from the mid-exponential growth phase (OD_600 _of 0.4) by centrifugation. The bacterial pellets were washed with 10 ml of THY and resuspended in 1 ml of THY. To prepare ^55^Fe^3+ ^ferrichrome working solution, 4.7 nmole ^55^FeCl_3 _was incubated with 14.1 nmole Fe-free ferrichrome A in 50 μL of Tris-HCl for 10 min, and the complex was diluted with 22 ml of THY. The 1-ml bacterial suspensions were mixed with 3 ml of the ^55^Fe^3+ ^ferrichrome solution (final Fe^3+ ^ferrichrome concentration 0.16 μM) to initiate the uptake process and rotated in a 10-ml tube from end to end at room temperature. A triplet of 0.2 ml samples were taken from each mixture at the indicated times, and bacteria were immediately pelleted and washed twice with 0.4 ml of THY. For uptake under growth conditions, 0.16 μM ^55^Fe^3+ ^ferrichrome was added into the cultures of wild-type, *ftsB*, and *ftsC *strains at early exponential or mid-exponential growth phase. A triplet of 1-ml culture samples were taken at the indicated times after the ^55^Fe^3+ ^ferrichrome addition and treated as described above. The pellets obtained were resuspended in 0.2 ml of THY and mixed with 3 ml of scintillation liquid. ^55^Fe^3+ ^radioactivity associated with the bacteria was measured as described above.

### FtsB and MtsA production

Production of FtsB or MtsA in wild-type and mutant strains was monitored by Western blotting analysis. To prepare samples for the analysis, bacteria were harvested from cultures at the indicated volumes in exponential phase. The bacterial pellets were washed twice with 1.3 ml of PBS, resuspended in 100 μl of PBS, and treated with 200 units of mutanolysin at 37°C for 2 h. The samples were briefly sonicated and mixed with equal volume of 2x SDS-PAGE loading buffer. Proteins in 10 μl of the samples were resolved by SDS-PAGE and transferred to nitrocellulose membranes. The proteins were detected by Western blotting using specific mouse antiserum as previously described [36].

### Other procedures and measurements

Chromosomal DNA was isolated with the Puregene DNA Isolation kit (Gentra Systems, Minneapolis, MN). Sequence data were obtained with an ABI 310 automated DNA sequencer (Applied Biosystems, Inc., Foster City, CA). Absorbance and optical spectra were obtained with a SPECTRAmax 384 Plus spectrophotometer (Molecular Devices, Sunnyvale, CA). Protein concentrations were determined with the modified Lowry protein assay kit purchased from Pierce (Rockford, IL) with bovine serum albumin as a standard. Intensities of the bands in Western blotting analysis were determined using an AlphaImager 2000 Documentation & Analysis System (Alpha Innotech Corp.).

## Authors' contributions

TSH carried out the ferric ferrichrome binding assay and the characterization of the GAS isogenic mutants and participated in writing the Methods section. ML generated the GAS isogenic mutants. MJM constructed the plasmid for complementing the *ftsB *mutant. BL designed the study and drafted the manuscript.

## Figures and Tables

**Table 1:** Primers used in gene inactivation and confirmation

**Primer**	**Sequence (5'-3')**	**Purpose**
1	ACCATGGCTATGCCTCAAATTGCTGG	*ftsB inactivation*
2	ACCATGGTTTTCCAATCTTTTAACCAC	
3	ACCATGGAGCTTATGTGTTGCTATTTAC	*ftsC inactivation*
4	ACCATGGATAGCTCCTGCTAAGACAAG	
5	ACCATGGTCTTTTAGTAGCTTGTTCGTC	*mtsA inactivation*
6	ACCATGGTTCATTTCGACAAGAGGCTG	
7	AACTCTACTATTAACACTCTG	confirmation of *ftsB *inactivation
8	GCAACGACGACATAATCACC	
9	GTGTCTCGAGCGTTTTTGC	confirmation of *ftsC *inactivation
10	CAATATAGCCGATCATTTG	
11	ATGAGCCTCATTTTGGGTGC	confirmation of *mtsA *inactivation
12	GCCACATAAGCTTTTAGGTTC	
13	AGAATTTTGTTAGCAGTTCG	*aad*-specific, paired with primer 7, 9, or 11 for inactivation confirmation
14	CTTTGAGTGAGCTGATACCG	pFWaad-specific, paired with primer 8, 10, or 12 for inactivation confirmation
